# Rational Design of a Triple Reporter Gene for Multimodality Molecular Imaging

**DOI:** 10.1155/2014/605358

**Published:** 2014-04-07

**Authors:** Ya-Ju Hsieh, Luen Hwu, Chien-Chih Ke, Skye Hsin-Hsien Yeh, Chien-Feng Lin, Fu-Du Chen, Hsin-Ell Wang, Kang-Ping Lin, Ran-Chou Chen, Ren-Shyan Liu

**Affiliations:** ^1^Department of Medical Imaging and Radiological Sciences, Kaohsiung Medical University, No. 100, Shih-Chuan 1st Road, Kaohsiung 80708, Taiwan; ^2^Molecular and Genetic Imaging Core/Taiwan Mouse Clinic, National Comprehensive Mouse Phenotyping and Drug Testing Center, No. 201, Section 2, Shih-Pai Road, Taipei 11217, Taiwan; ^3^Institute of Clinical Medicine, National Yang-Ming University, No. 155, Section 2, Linong Street, Taipei 11221, Taiwan; ^4^Department of Biomedical Imaging and Radiological Sciences, National Yang-Ming University, No. 155, Section 2, Linong Street, Taipei 11221, Taiwan; ^5^Department of Education and Research, Taipei City Hospital, No. 145, Zhengzhou Road, Datong District, Taipei 10341, Taiwan; ^6^Biophotonic and Molecular Imaging Research Center, National Yang-Ming University, No. 155, Section 2, Linong Street, Taipei 11221, Taiwan; ^7^Nuclear Medicine Department and PET-CT Center, Shunang Ho Hospital Ministry of Health and Welfare, Taipei Medical University, No. 291, Zhongzheng Road, Zhonghe District, New Taipei City 23561, Taiwan; ^8^Center for Teaching and Learning Resources, Chinese Culture University, No. 55, Hwa-Kang Road, Taipei 11114, Taiwan; ^9^Department of Electrical Engineering and Holistic Medical Device Development Center, Chung Yuan Christian University, No. 200, Chung Pei Road, Chung Li City 32023, Taiwan; ^10^National PET/Cyclotron Center and Department of Nuclear Medicine, Taipei Veterans General Hospital, No. 201, Section 2, Shih-Pai Road, Taipei 11217, Taiwan

## Abstract

Multimodality imaging using noncytotoxic triple fusion (TF) reporter genes is an important application for cell-based tracking, drug screening, and therapy. The firefly luciferase *(fl)*, monomeric red fluorescence protein *(mrfp)*, and truncated herpes simplex virus type 1 thymidine kinase SR39 mutant *(ttksr39)* were fused together to create TF reporter gene constructs with different order. The enzymatic activities of TF protein in vitro and *in vivo* were determined by luciferase reporter assay, H-FEAU cellular uptake experiment, bioluminescence imaging, and micropositron emission tomography (microPET). The TF construct expressed in H1299 cells possesses luciferase activity and red fluorescence. The tTKSR39 activity is preserved in TF protein and mediates high levels of H-FEAU accumulation and significant cell death from ganciclovir (GCV) prodrug activation. In living animals, the luciferase and tTKSR39 activities of TF protein have also been successfully validated by multimodality imaging systems. The red fluorescence signal is relatively weak for *in vivo* imaging but may expedite FACS-based selection of TF reporter expressing cells. We have developed an optimized triple fusion reporter construct *DsRedm-fl-ttksr39* for more effective and sensitive *in vivo* animal imaging using fluorescence, bioluminescence, and PET imaging modalities, which may facilitate different fields of biomedical research and applications.

## 1. Introduction

Multiple molecular imaging techniques have been used not only to monitor complex biological processes both spatially and temporally but also to study the transplanted cell trafficking, long term monitoring of disease (i.e., cancer progression), and therapeutic effectiveness in living animals [1, 2]. These techniques have accelerated the translation of* in vitro* based drug discovery studies to* in vivo* imaging-based preclinical and clinical applications [3]. Several noninvasive imaging modalities such as positron emission tomography (PET), single photon emission computed tomography (SPECT), magnetic resonance imaging (MRI), and optical imaging are available for real-time repetitive imaging of reporter gene expression in living subjects [1–4]. Optical imaging is quick and cost-effective and requires no radioisotopes for validating different reporter systems. However, it does not provide optimal tomographic information and quantitative data as compared to 3D radionuclide imaging techniques (i.e., PET, SPECT) [5]. A dual or triple fusion reporter gene construct harboring various fluorescent proteins (i.e., enhanced green fluorescent protein (*egfp*), red fluorescent protein (*rfp*), and different luciferase genes (i.e., firefly luciferase (*fl*), renilla luciferase (*rl*), and herpes simplex virus type 1 thymidine kinase (*tk*) gene)) can combine the advantage of fluorescence, bioluminescence, and radionuclide imaging techniques and overcome the shortcomings of each imaging modality [6].

The* tk-gfp* dual fusion gene allows for microscopic and whole-body fluorescence imaging as well as PET imaging [7]. Another fusion gene, the* tksr39-rl* (*tksr39* is a mutant version of* tk *[8]), was also validated in living mice bearing transduced tumor xenografts by microPET and bioluminescence imaging (BLI) [9]. A triple fusion (TF) reporter gene, truncated* tk-egfp-fl*, was constructed to visualize the TF-transduced cells in living animals by optical, fluorescent, and nuclear imaging modalities [10]. However, the carboxyl-terminal (C-terminal) region of* tk* is involved in the enzymatic catalysis profoundly [11] and fusion of the partner gene such as* fl* gene to the C-terminal region of* tk* would lead to a decreased TK activity (note that* tk* refers to the gene and TK to the protein) [9]. The configuration of* tk-egfp-fl* TF reporter gene is not favorable due to severe influence on TK folding and enzymatic function from the downstream large bulky EGFP-FLUC fusion protein. Several other TF genes had also been constructed and validated, that is,* rl-egfp-ttksr39* [12],* rl-mrfp-ttksr39*, and* fl-mrfp-ttksr39 *[3, 13] (*mrfp*: monomeric red fluorescent protein;* ttksr39*: truncated* tksr39*). Because of the intrinsic autofluorescence problem in the living animals, the* egfp*-harboring TF gene constructs were not chosen for* in vivo* imaging applications. In contrast, the efficacy of TF gene construct harboring* rfp* gene in the 293T human embryonic kidney cells or A375M human melanoma cells has been demonstrated in living mice with microPET and optical imaging systems [3]. The* fl-mrfp-ttksr39* reporter gene has also been applied in stem cell research. In 2006, Cao et al. developed murine embryonic stem cells (ESCs) stably expressing human ubiquitin-C (UBC) promoter-driven TF fusion gene and demonstrated that kinetics of ESC survival, proliferation, and migration in living mice could be monitored using multimodality imaging systems [14, 15]. Using lentiviral-mediated transduction, the modified myeloproliferative sarcoma virus (mnd) promoter-driven TF gene can be stably expressed in human mesenchymal stem cell (MSC) for* in vivo* monitoring of the efficacy of stem cell transplantation [16]. It is noteworthy that there are no experimental data using stem cells transduced with an* fl-mrfp-ttksr39* gene construct driven by a constitutive cytomegaloviral (CMV) promoter. Several studies have reported epigenetic silencing of CMV promoter after longitudinal passage of stem cells [16, 17]. Thus, we speculated whether the lentiviral-mediated overexpression of* fl-mrfp-ttksr39* TF driven by the CMV promoter would induce aberrant characteristics or alter differentiation capacity of stem cells.

For drug screening and validation, a less cytotoxic and highly sensitive reporter gene that faithfully reflects the drug effectiveness and eludes the cytotoxic effect of reporter gene product is required. Furthermore, for cell therapy and trafficking, it is also necessary to adopt a less cytotoxic reporter gene to minimize the cellular stress from the aggregation-prone reporter gene product in cell labeling. However, considering the detection sensitivity and limitation, the reporter gene was usually driven by a strong promoter to increase the reporter gene expression and consequently the aggregation-prone reporter gene products accumulated in the transfected cells and ultimately resulted in cellular stress and cytotoxic effect that hampered the cell growth. Previous reports indicated that the* rl-mrfp-ttksr39* or* fl-mrfp-wt.ttk* (*wt.ttk*; truncated wild type tk) is the best configuration of TF gene constructs for multimodality imaging techniques, if the* rl* or* fl* are chosen for TF [3, 13]. To best preserve the TK activity for radionuclide imaging the* ttksr39* should be combined with the* rl-mrfp* component and the* wt.tk* should be combined with the resting* fl-mrfp* component. The enzymatic activity of* ttksr39* or* wt.tk* could be significantly influenced by fusion with different fusion partners. Furthermore, the intracellular localization of TK-harboring fusion proteins changes significantly while different fusion partner is in-frame fused to* tk* gene or the same fusion partner is in-frame fused to* tk* at N- or C-terminal. The GFP-TK fusion protein is exclusively and homogenously expressed in the nuclei of the transduced cells, whereas TK-GFP exists as pancellular localization but the majority of proteins are in the nucleus with the massive aggregated form of nuclear bodies [10, 18]. Such intracellular localization of TK-containing fusion protein will result in a reduced enzymatic activity of TK and most likely cause severe cytotoxic effects to cells due to the reporter protein aggregation in the cell nucleus [19, 20]. Because of the advantage of TF reporter construct in the research, we attempted to establish cancer cell clones stably expressing CMV driven* fl-DsRedm-ttksr39* TF gene construct. However, our unpublished studies demonstrated that the fast chromophore maturation of TF is similar to primitive tetrameric DsRed protein suggesting that the TF protein in transfected cells might exist in a tetrameric form. The cell clones only transiently expressed the TF fusion protein and the survived cells did not proliferate after G418 selection and failed to establish clonal populations. Thus, we postulated that the* fl-DsRedm-ttksr39* protein may be toxic to cells. Since the order of fusion genes in TF determines the mRNA structure that may influence ribosomal accessibility or mRNA stability, we aimed to construct a new TF reporter gene with optimal configuration for multimodality imaging, the p3H (*DsRedm-fl-ttksr39*), and to compare this novel TF gene with other two TF genes, the p3G (*rl-DsRedm-ttksr39*) and p3R (*fl-DsRedm-ttksr39*).

## 2. Experimental Procedures

### 2.1. Construction of Triple Fusion Reporter Gene

The* rl-DsRedm-ttksr39* (p3G) and* fl-DsRedm-ttksr39* (p3R) were constructed as described by Ray et al. [3, 12]. The novel triple fusion reporter plasmid,* DsRedm-fl-ttksr39* (p3H), was constructed essentially as described by Ray et al. [3, 12]. Briefly, the monomeric DsRed expression plasmid,* pDsRed-Monomer -C1*, driven by CMV enhancer/promoter, was purchased from Clontech (BD science, Inc., USA). The* pDsRed-Monomer-C1* digested with restriction enzyme of BamH I and Xba I (New England Biolabs, Inc., USA) was purified from 1% agarose gel by PCR/Gel Extraction kit (Geneaid Inc., Taiwan) and used as cloning vector. The truncated tksr39 gene was amplified from the plasmid pttksr39 (generous gifts from Professor FD Chen, TransWorld University, Taiwan) by polymerase chain reaction (PCR) with 5′-end primer ttkUp/BamHI (5′-CAA GAC GGA TCC TCT GGT AAA ATG CCC ACG CTA CTG C-3′), 3′-end primer ttkDn-XbaI (5′-GTA TTC TCT AGA TCA GTT AGC CTC CCC CAT C-3′), and the proof-reading KOD Taq DNA polymerase (Novagen Inc., USA). The ttksr39 PCR products were purified and subjected to restriction enzyme of BamH I and Xba I (New England Biolabs, Inc., USA) digestion and then the purified ttksr39 insert DNAs were ligated with BamH I-XbaI digested* pDsRed-Monomer-C1* vector by T4 DNA ligase (New England Biolabs, Inc., USA) generating a* DsRedm-ttksr39* dual fusion reporter genetic construct. The* DsRedm-ttksr39* plasmid was digested with restriction enzyme of EcoR I and Sal I (New England Biolabs, Inc., USA) and used for a cloning vector. The* fl* gene from the pGL3 basic plasmid (Promega Corporation, Madison, WI, USA) was amplified by PCR using the same 5′-end primer FLUCUp-EcoRI 5′-AGC ATC GAA TTC TGA GGA CGC CAA AAA CAT AAA G-3′, the 3′end primer FLUCDn-SalI 5′-CTA GTA GTC GAC AGC AAT CTT TCC GCC CTT CT-3′, and the proof-reading KOD Taq DNA polymerase (Novagen Inc., USA). After purification, the* fl* PCR products were digested with restriction enzyme of EcoR I and Sal I and then were ligated with the EcoR I-Sal I digested* DsRedm-ttksr39* cloning vector to create the* DsRedm-fl-ttksr39* triple fusion reporter genetic construct. To create the* DsRed1-ttksr39* construct, the* pDsRed1-C1 *plasmid was digested with Age I and Bam HI, and the* DsRed1* gene was purified from agarose gel electrophoresis and was used as DNA inserts. The* DsRedm-fl-ttksr39* (p3H) plasmid was digested with the same restriction enzymes to remove the* DsRedm-fl* DNA fragment and was used as a vector for cloning DsRed1 gene. The DNA coding sequences of all constructs were verified by DNA sequencing service (Misson Biotech, Inc., Taiwan) using ABI model 3730 DNA sequencer.

### 2.2. Cell Culture

The H1299 nonsmall cell lung cancer cells were maintained in RPMI 1640 growth medium supplemented with 10% fetal calf serum at 37°C. Cells were incubated in a humidified incubator at 37°C and 5% CO_2_ : 95% air.

### 2.3. Cell Transfection and Luciferase Reporter Assays

Cells were seeded into 12-well plates (Orange Inc., USA) at a concentration sufficient to give 80–90% confluency on the day of transfection, typically 8 × 10^4^ cells/well, and cultured overnight. In all, 1.5 *μ*g trifusion reporter plasmids were cotransfected with 0.5 *μ*g of* CMV-rl*, in which the rl was driven by CMV promoter, as an internal control for transfection efficiency using Lipofectamine 2000 transfection reagent (Invitrogen, USA) according to the manufacturer's instructions. Briefly, 2 *μ*g of plasmid DNA and 6 *μ*L of Lipofectamine 2000 transfection reagent diluted in serum-free culture medium (Gibco, USA) were used for each transfection. After a 48-hr incubation, cells were analysed for both firefly and renilla luciferase expression using the Dual Luciferase Assay (Promega, USA). All transfections were carried out in triplicate and normalized by the internal control and amounts of protein. Results presented are the means of at least three independent experiments.

### 2.4. Cellular Uptake Studies

The H1299 cells were cotransfected with reporter gene (p3H, p3R, p3G,* CMV-ttksr39*, and* DsRed1-ttksr39*; 1.8 *μ*g of each construct) and* CMV-egfp* (0.2 *μ*g). The transfected cells (1.0 × 10^5^ cells) were plated in 12-well dishes. Twenty-four hours after ^3^H-FEAU (1.48 × 10^3^ Bq/mL [0.4 *μ*Ci/mL]) was added in each well and incubated at 37°C for 2 h, cells were washed with cold phosphate-buffered saline (PBS) and lysed by protein extraction reagent (CytoBuster, Novagen, USA), and the radioactivity of each well was measured using beta counter (Perkin-Elmer, USA). The same wells were used to determine the total protein. Because the transfection efficiency of different gene constructs in H1299 cell lines may not be identical, the results were normalized by measuring EGFP expression levels. All results were expressed as the net accumulation of the probe in dpm of cells/dpm of medium/EGFP/*μ*g total protein ± standard error (SE).

### 2.5. GCV-Mediated Growth Inhibition Studies

Twenty-four hours after transfection with* CMV-fl*, p3H, p3G, p3R, or* DsRed1-ttksr39* plasmids, the transfected cells were reseeded in 96-well flat-bottom microtiter cell culture plates and incubated overnight. Cells were then treated with 300 *μ*L of fresh medium in the presence of varying concentrations of ganciclovir (GCV) (Roche, Germany) for five days at 37°C. The MTT [3-(4,5-dimethyl-thiazol-2-yl)-2,5-diphenyl tetrazolium bromide] (Sigma, USA) was used to treat the cells for 2 h at 37°C. To quantify the cell viability, the formazan products solubilized by DMSO (Sigma, USA) were measured by spectrophotometer (Bio-Rad, USA) with 570 nm and 630 nm of wavelength.

The percentage of cell survival was calculated as

(1) $$\begin{array}{c} \text{Cell}\;\text{viability}=\frac{B}{A}\;100\%, \end{array}$$

where *A* is the absorbance from the cells incubated with various concentrations of GCV and *B* is the absorbance from the cells incubated with the medium containing various concentrations of GCV.

### 2.6. *In Vivo* MicroPET and Cooled CCD Optical Imaging

The nu/nu nude mice (6~8-week-old, male) were purchased from National Laboratory Animal Center, Taiwan. About 8 × 10^6^ H1299 cells transfected with TF plasmids were subcutaneously injected into the shoulders and legs of the mice. Forty-eight hours posttransfection, each mouse was placed in the black chamber of CCD camera IVIS 50 (Perkin Elmer, USA) equipped with a halogen light source, an excitation filter at 500–550 nm, and an emission filter at 575–650 nm to acquire a whole body imagefor 1 s. For bioluminescence imaging, the mice were injected with D-luciferin (Promega, USA) intraperitoneally for analysis of* fl* gene expression (*n* = 4) and injected later with coelenterazine (Promega, USA) through tail vein for analysis of* rl* gene expression. Subsequently, static images were obtained from the same anesthetized animals intravenously injected with 0.13 MBq (35 *μ*Ci) of ^18^F-FEAU (100 *μ*L) by using microPET R4 (Concord Microsystems, USA) (*n* = 4). The bioluminescence and fluorescence signals were obtained by a CCD camera thermoelectrically cooled to −70°C. Bioluminescence signals were displayed in pseudocolors and superimposed on the photographic image using Xenogen's Living Image software. Both bioluminescence and fluorescence signals were recorded as maximum photons/second/centimeter^2^/steradian (photons/ sec/cm^2^/sr). Regions of interest (ROIs) in microPET image were drawn over the tumor and the ROI counts were converted to percentage of injected dose per gram (%ID/g) using filtered back projection [3].

### 2.7. Immunofluorescence Staining

H1299 transfected cells were seeded onto poly-(lysine) coated coverslips and fixed with 4% paraformaldehyde. Cells permeabilized with 0.1% of Triton X-100 were blocked in 4% BSA/PBS and subsequently probed with primary antibody anti-HSV tk Ab (a generous gift from Professor Juri Gelovani, MD Anderson Cancer Center, TX, USA) followed by goat anti-mouse IgG antibody conjugated to FITC (Santa Cruz, USA). The 4′,6-diamidino-2-phenylindole (DAPI) was dropped onto the sections to stain the nuclei. Fluorescent signals from DsRed- or HSV1 tk-expressed cells were obtained using confocal microscopy (Leica TCS SP5, Germany).

### 2.8. Statistical Analysis

The mean and standard error values were calculated for each group and experimental. The 2-tailed Student's *t*-test and two-way ANOVA were used for statistical analysis using SPSS 15.0 (SPSS, Inc., IL, USA). Statistical significance was defined as *P* < 0.05.

## 3. Results

### 3.1. *In Vitro* Demonstration of the Efficacy of Triple Fusion Reporter Gene

**Figure 1:**
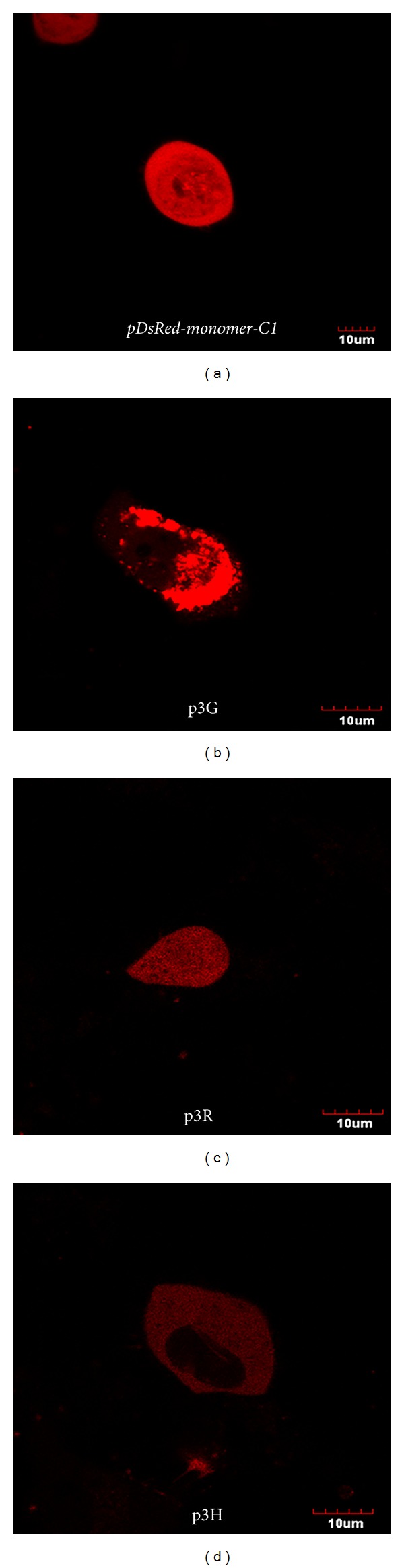
Red fluorescence photomicrographs of H1299 cells expressing* pDsRed-Monomer-C1*, p3G, p3R, and p3H gene constructs. Bars for the fluorescence micrographs represent 10 *μ*m.

**Figure 2:**
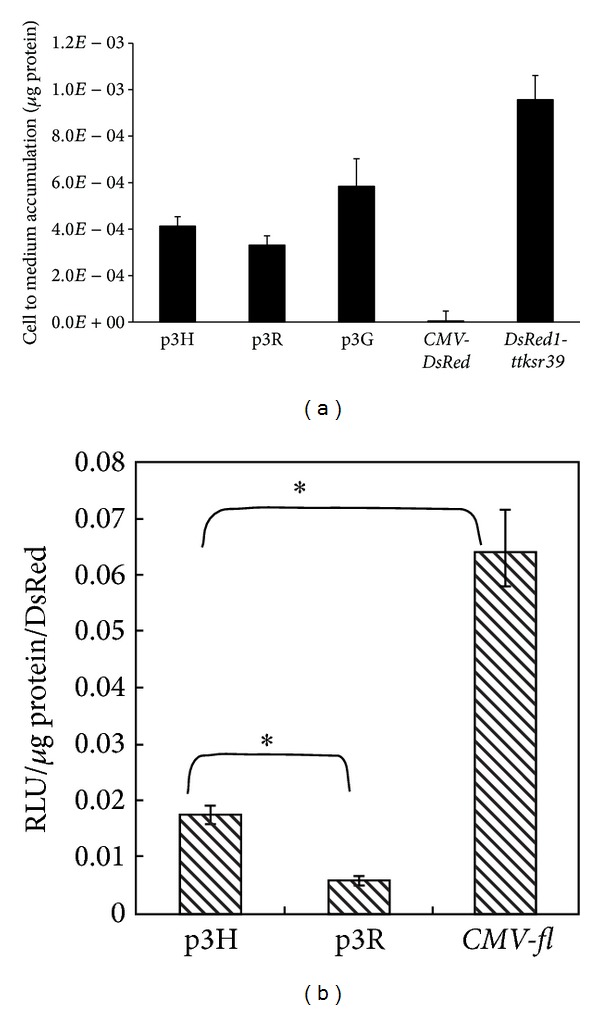
The firefly luciferase and tTKSR39 activity exhibited by H1299 cells transiently transfected with different gene constructs. The H1299 cells were cotransfected with* CMV-rl* and* fl*-harboring gene constructs. The tTK activity normalized with CMV-EGFP was expressed as cell to medium accumulation/g protein (a). Values for FLUC activity was normalized with RLUC activity and expressed as relative light units (RLU)/g protein/RLUC activity (b). All the experiments (*n* = 4) were done in triplicate. *P < 0.05.

Plasmid DNA of three different TF vectors (p3G, p3R, and p3H) has been used to transiently transfect the H1299 cells, and* pDsRed-Monomer-C1* (BD Science, Clontech, USA) was used for positive control. The transfected cells were first visualized by fluorescence microscopy for monomeric DsRed activity and further assayed for either RLUC or FLUC and tTKSR39 activities. Fluorescence microscopy of transfected cells demonstrated homogenous distribution of positive control protein (*pDsRed-Monomer-C1*) throughout the nucleus and cytoplasm (Figure 1). However, fluorescence in p3H (*DsRedm-fl-ttksr39*) transfected cells was predominantly and uniformly distributed in the cytoplasm with slight fluorescence detectable in the cell nucleus. The majority of cells expressing p3G (*rl-DsRedm-ttksr39*) gene demonstrated tightly packed red fluorescent aggregates mainly in the cytoplasm. Only a small amount of fusion protein appeared in the nucleus. The cells transfected with p3R (*fl-DsRedm-ttksr39*) gene demonstrated a similar distribution pattern of TF fusion protein as positive control, with less aggregation in the cytoplasm and nucleus as compared with the p3G-transfected cells. Among all gene constructs used in this study, the p3G-transfected cells demonstrated higher red fluorescence intensity, as compared to* pDsRed-Monomer-C1* positive control, whereas cells expressing either p3R or p3H gene exhibited relatively lower fluorescence intensity. Curiously, the red fluorescent signal from the p3G and p3R expressing cells could be observed already overnight posttransfection, as compared to pDsRed-Monomer-C1 and p3H transduced cells, of which the fluorescence was not detectable until 36 hrs posttransfection (data not shown). This phenomenon suggests that p3G and p3R TF reporter protein has faster chromophore maturation rate than that of monomeric DsRed protein. The faster maturation rate is reminiscent of the tetrameric DsRed protein trait. To assess the tTKSR39 enzyme activity, H1299 cells were transiently transfected with TF reporter vectors along with positive control (*CMV-ttksr39*) and negative control (*CMV-DsRed*). The uptake of ^3^H-FEAU in p3G-transfected cells was the highest among the three TF reporter vectors (*P* < 0.05), about 33% of positive control (Figure 2(a)). In* fl*-harboring TF vectors, the p3H-transfected cells accumulated more ^3^H-FEAU than the p3R-transfected cells (23% versus 19%), indicating that higher tTKSR39 activity is achievable using the p3H vector (*P* < 0.05). Also, we constructed a dual reporter gene (*DsRed1-ttksr39*) as another positive control. In the cells transfected with* DsRed1-ttksr39* gene, a 46% decrease in ^3^H-FEAU uptake was observed, as compared with the positive control (*CMV-ttksr39*). However, the tTKSR39 activity in the DsRed1-ttksr39 construct was still higher (54%) than all three TF vectors. Further, luciferase enzyme assay was used to compare the FLUC activity achieved by p3H and p3R vectors; the* CMV-fl* was used as a positive control for reporter gene expression. The FLUC activity was normalized by measuring the activity of cotransfected* CMV-rl* using dual-luciferase reporter assay system (http://www.promega.com/tbs/). The highest FLUC activity was observed in cells transfected with* CMV-fl* (Figure 2(b)). The p3H vector retained about 27% of the FLUC activity measureable with positive control and exhibited threefold higher activity than the p3R vector (*P* < 0.05).

### 3.2. Subcellular Localization of Different Triple Fusion Proteins

**Figure 3:**
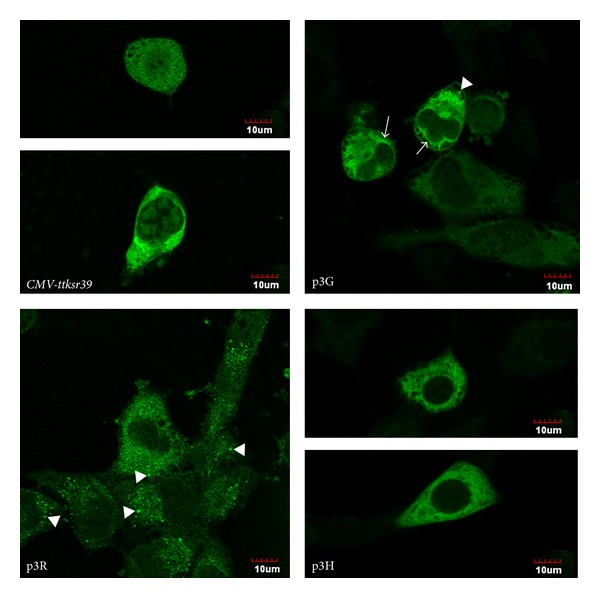
Immunofluorescence staining for TKSR39 protein expressed by cells transfected with* CMV-ttksr39*, p3G, p3R, or p3H gene constructs. Arrow heads indicate aggregated proteins of p3G and p3R in cytoplasm. Arrows indicate peri-nuclear distribution of p3G proteins. Bars for the fluorescence micrographs represent 10 *μ*m.

Immunofluorescence staining of transfected cells demonstrated distinct differences in subcellular localization of native tTKSR39 and fused tTKSR39 (p3G, p3R, and p3H) proteins. The single tTKSR39 protein exhibited uniform distribution in the nucleus and cytoplasm (Figure 3). Fusion of ttksr39 with p3G, p3R, and p3H genes resulted in predominant cytoplasmic distribution of fusion proteins (Figure 3). This effect was more pronounced in p3H-expressing cells than p3G- and p3R-expressing cells. Moreover, the cells transfected with p3R showed a tendency to form small aggregation in the perinuclear region and the cytoplasm.

### 3.3. GCV-Mediated Cytotoxicity in Triple Fusion Reporter Gene-Transfected Cells

**Figure 4:**
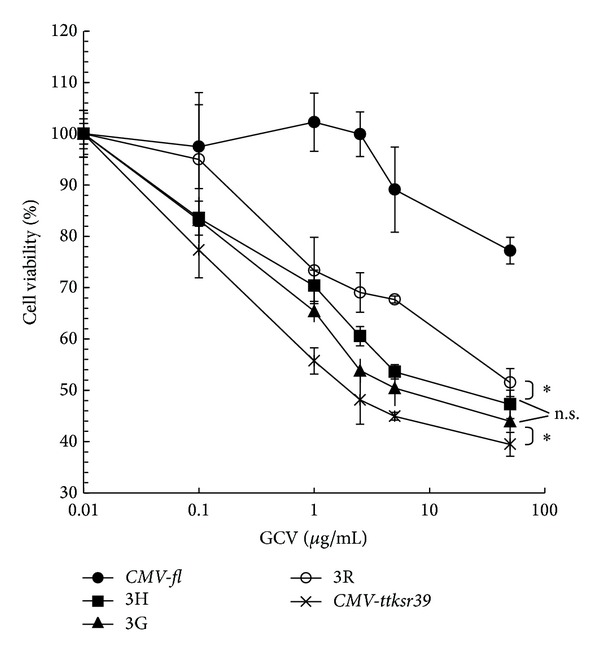
*In vitro* cytotoxicity effect of GCV in H1299 cells transfected with* CMV-fl* (●), p3H (■), p3G (▲), p3R (⚪), or* CMV-ttksr39* (**×**) constructs. Cells transfected with these constructs were incubated with various concentrations of GCV for 5 days followed by cell survival estimation using MTT. All the experiments (*n* = 4) were done in triplicate. *P < 0.05; n.s.: statistically nonsignificant.

Efficient tTKSR39 expression in the TF-transfected cells should facilitate the activation of GCV and subsequently enhance cell killing. Cells transfected with either* CMV-fl* or* CMV-ttksr39* were used as negative and positive controls, respectively. The slight decrease in cell viability observed in negative control was due to the nature toxicity of GCV to cells (Figure 4). Consistent with the results of* in vitro* ^3^H-FEAU accumulation studies, the positive control showed the highest level of tTKSR39 resulting in significant decrease in cell viability (Figure 4). In contrast, all three TF-transfected cell lines exhibited less pronounced GCV-induced cell death than the positive control cells; the cell death induced by GCV in p3R-transduced cells was the lowest among three TF vectors.

### 3.4. *In Vivo* Imaging of the Triple Fusion Reporter Gene Expression by Optical and Radionuclide Techniques

**Figure 5:**
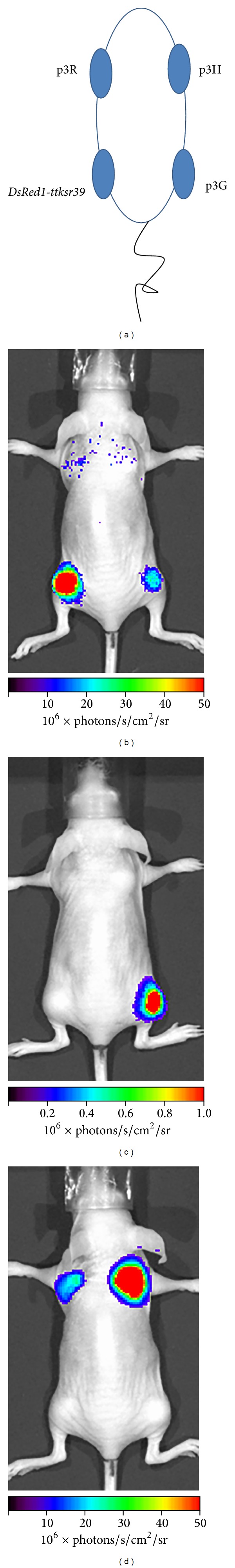
Demonstration of triple fusion gene vector in living subjects by optical imaging. Cells transiently expressing p3R, p3H, p3G, and* DsRed1-ttksr39* genes were implanted subcutaneously at four different sites of a nude mouse (a). Red fluorescence signals were detectable only in the regions implanted with* DsRed1-ttksr39*- and p3G-expressing cells (b). Bioluminescence signals resulted from RLUC were found in p3G-expressing cells (c). Different levels of bioluminescence signals resulted from FLUC were observed in p3H- and p3R-expressing cells (d).

**Figure 6:**
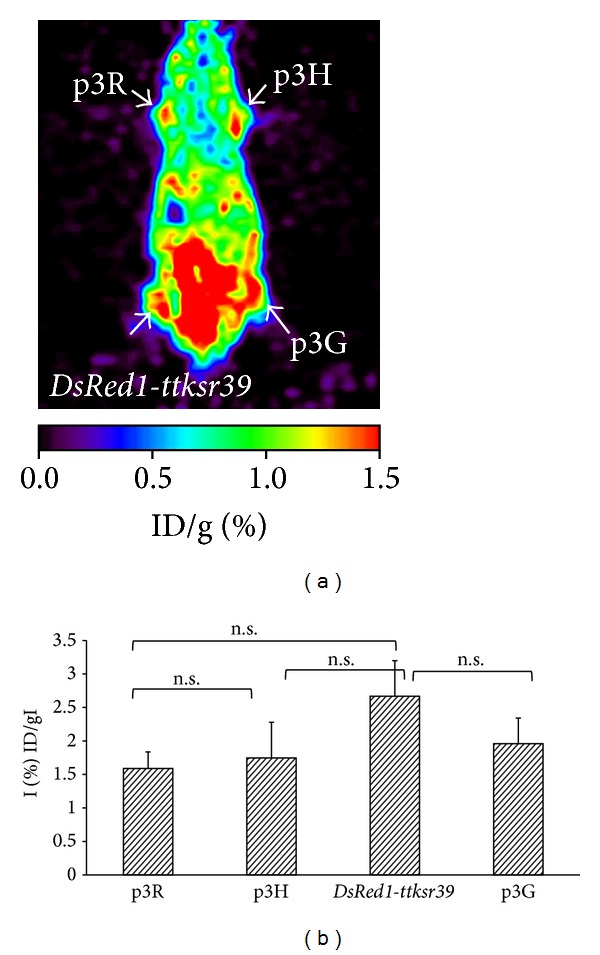
Demonstration of triple fusion gene vector in living subjects by microPET imaging. The same mouse demonstrated previously by optical imaging was then imaged by microPET using ^18^F-FEAU. Cells expressing the fusion reporter genes and* DsRed1-ttk* gene showed ^18^F-FEAU accumulation in the tumor (coronal section) (a). ROIs drawn over the sites of cell implantation of four mice were calculated and expressed as percentage of injected dose per gram of tissue (% ID/g). Each bar represents the mean ± SD (*n* = 4) (b). n.s.: statistically nonsignificant.

Subsequent to* in vitro* studies, we investigated whether the TF vectors would simultaneously and repeatedly express the three fusion proteins in living subjects and would be detectable using different imaging modalities. The H1299 cells transiently transfected with p3G, p3R, p3H, or* DsRed1-ttksr39* vectors were injected subcutaneously into the shoulders and legs of 6~8-week-old nude mice (Figure 5(a)). The red fluorescence was detected in tumors grown from p3G-transfected cells with the signal (80.8 ± 3 × 10^6^ photons/sec/cm^2^/sr) lower than that observed in positive control tumors established from cells expressing DsRed1-ttksr39 gene (225 ± 37 × 10^6^ photons/sec/cm^2^/sr) but was not detectable in tumors grown from p3R- or p3H-transfected cells (Figure 5(b)). The expression efficacy of* rl* reporter gene in tumors established from p3G-transfected cells was demonstrated by bioluminescence imaging (Figure 5(c)). The bioluminescence signal of RLUC (1.65 ± 0.55 × 10^6^ photons/sec/cm^2^/sr) was observed in tumors established from cells expressing p3G protein. Tumors expressing p3H protein showed 4-fold higher FLUC activity (211 ± 137 × 10^6^ photons/sec/cm^2^/sr) than tumors expressing p3R protein (50.4 ± 12.44 × 10^6^ photons/sec/cm^2^/sr) (Figure 5(d)). Because FEAU has a better substrate affinity to tTKsr39 enzyme than acycloguanosine FHBG [21] and the accumulation of FEAU in the gastrointestinal tract is lower than FHBG, we thus selected ^18^F-FEAU for monitoring of the* ttksr39* reporter gene expression in living subjects (Figure 6(a)). Tumors expressing* DsRed1-ttksr39* positive control gene showed 1.36-fold higher level of ^18^F-FEAU uptake (2.66 ± 0.26 %ID/g) than tumors expressing p3G TF gene (1.96 ± 0.38 %ID/g) (Figure 6(b)). A trend of decreasing ^18^F-FEAU uptake (p3G > p3H > p3R) in all three TF vectors was observed. However, the differences in ^18^F-FEAU uptake between each tumor did not reach statistical significance.

## 4. Discussion

Genetic fusion reporter systems have greatly facilitated studies on the regulation of gene expression, as well as protein localization and function using multiple imaging modalities. Several triple fusion (TF) reporter genes harboring a bioluminescence reporter genes (i.e.,* rl *or* fl*), fluorescence reporter genes (i.e.,* mrfp* or* egfp*), and a radionuclide imaging reporter gene (*ttksr39* or* wt.tk*) have been utilized for* in vivo* imaging of small animals. Our primary interest was to develop TF reporter gene constructs for research applications in cancer, stem cell, and regenerative medicine. However, we have observed that several types of stem cells lost their proliferation ability and showed morphological abnormalities after transfection with p3R developed by Ray et al. [17]. These problems have been resolved in a novel TF gene construct-p3H by changing the orientation of* fl* and* DsRedm* genes in the expression cassette. The C-terminal region of TK is essential for maintaining nucleoside phosphorylation activity of this enzyme [11]. Moreover, the N-terminal tTKSR39 fusion proteins are susceptible to enzymatic cleavage [9]. Therefore, we placed the* ttksr39* gene at the carboxyl end of a TF gene construct. The different subcellular distribution pattern of monomeric DsRed or TK in all three TF gene constructs demonstrated that the localization of fusion protein can be influenced not only by the nature of different fusion partner proteins but also by their order in the fusion.

It has been established that chromophore maturation rate and brightness of monomeric DsRed protein are lower than dimeric or tetrameric form [22, 23]. In the current study, fluorescence microscopy demonstrated that chromophore maturation rate in p3G- and p3R-transfected cells (<24 hrs) was faster than that in p3H-transfected cells (>24 hrs), which caused a significant condensation of TF protein in the cytoplasm. One possible explanation of this observation is that the sequence of proteins in p3G and 3R is more prone to formation of DsRed beta-barrel structure, as compared to p3H. Previous studies have also pointed out that due to high levels of reporter gene expression mediated by CMV promoter, the transfected cells may exhibit growth defects [24, 25] and detach from the growth surface [24]. Such cytotoxicity was typical of high levels of RFP expression that causes aggregation of this protein [24, 26]. Significant protein aggregates will lead to the impairment of ubiquitin-proteasome system (UPS) that plays an important role in regulating cell division and apoptosis and may cause cell cycle arrest in G2/M [19, 27]. This mechanism may explain at least in part the failure of our previous efforts to establish stable clones of transduced tumor cells using p3G or p3R TF constructs. By using split synthetic renilla luciferase complementation-based bioluminescence assay, Degrève et al. demonstrated the homodimerization of HSV1-TKSR39 [28]. If the tTKSR39 fusion part in p3G or p3R forms homodimer more easily than p3H, the DsRedm fusion part may also form dimeric or tetrameric form resulting in faster chromophore maturation and obvious protein aggregation. In contrast, in p3H the FLUC positioned between DsRed and tTKSR39 may act as a structural barrier to avoid dimerization or tetramerization mediated by tTKSR39. However, as the result of such positioning of RFP in p3H construct, the brightness of RFP fluorescence is relatively lower than that in p3G and p3R.

Previous crystal structure studies revealed that FLUC has a large N-terminal domain (residues 1–436) and a small C-terminal domain (residues 440–550) [29]. During the enzyme-substrate reaction, the distance between N- and C-terminal needs to be close enough to sandwich the substrate [30]. Previous studies indicated that not only the C-terminal domain of FLUC is indispensable for efficient coupling of adenylation and oxidation steps [21] but also the extreme N-terminal amino acid sequence of FLUC is important in thermal stability and proper conformation of this enzyme [31]. In fact, the N-terminal amino acids from residues 1 to 11 are not directly involved in the active site of FLUC, which is located in the cleft formed by N- and C-terminal domains [21]. The FLUC activity has been shown to be 50-fold higher in cells expressing the* wt.tk-fl* fusion gene, as compared to cells expressing the* fl-wt.tk* fusion gene [32]. To test the effect of N-terminal amino acid modification on the FLUC enzyme activity, the GAL4 DNA binding domain was fused with the N terminus of* fl*, and the results showed that the enzymatic activity of FLUC from GAL4-FLUC fusion protein is as normal as in the nonfused protein [31]. The N terminus of FLUC seems to be more tolerant of fusions than the C terminus. Therefore, we hypothesized that changing the sequence of genes in the* fl-mrfp* fusion to* mrfp-fl* may improve the enzymatic activity of FLUC. The results of our studies demonstrated a 3-fold improvement of FLUC enzymatic activity in p3H-expressing cells, as compared to the p3R-expressing cells, which supports our hypothesis. Another probable reason for impaired FLUC activity is that the close proximity of the bulky DsRedtTKSR39 to FLUC may increase the distance between C- and N-terminal domains of FLUC and interfere with the active site formation and/or function [30, 31]. As expected, the functional activity of FLUC was partially preserved in both p3H and p3R TF gene constructs.

The truncated tksr39 we choose for one partner of our TF gene construct lacks the first 45 amino acids and is designed to disrupt its nuclear localization signal (NLS) and a putative cryptic testicular-specific promoter, which causes sterility in transgenic mouse males [28, 33]. TF constructs harboring this truncated protein exhibit pancellular distribution of the tTKSR39 reporter protein, whereas the wild type TK (wt.TK) localizes predominantly to the cell nucleus and results in improved total cellular enzymatic activity [18]. In addition, the TKSR39 exhibits increased sensitivity towards different acycloguanosine analogues when compared with wt.TK [3]. The TKSR39 has been extensively studied as a reporter gene for noninvasive imaging with PET using different radiolabeled acycloguanosine analogues as reporter probes, including [^14^C]1-(2′-fluoro-2′-deoxy-d-arabinofuranosyl)-5-methyluracil (^14^C-FMAU), [^3^H]2′-fluoro-2′-deoxyarabinofuranosyl-5-ethyluracil (^3^H-FEAU), [^14^C]2′-fluoro-2′-deoxy-*β*-d-arabinofuranosyl-5-iodouracil (^14^C-FIAU), and [^3^H] penciclovir (^3^H-PCV) [34]. In the current study, the immunofluorescence images of tTKSR39 protein demonstrated that the p3H-expressing cells have more cytoplasmic localization of TF protein than the p3R-expressing cells indicating that higher tTKSR39 activity is preserved in the p3H construct. Moreover, a significant aggregation of TF protein was observed in the cytoplasm and, especially, in the perinuclear regions of p3R-expressing cells. Such abnormal aggregation of p3R protein had a negative effect on the efficacy of GCV prodrug activating ability of tTKSR39 and resulted in decrease in GCV-induced cytotoxicity, as well as decreased the cellular uptake of ^3^H-FEAU, as compared to p3G or p3H reporter constructs. Furthermore, in mouse imaging studies we observed the red fluorescence signals in the implanted tumors established from p3G-transfected cells, albeit the RFP gene expression of p3G was significantly lower than the DsRed1-ttksr39 (positive control). This observation is consistent with previous reports demonstrating that* DsRed1* gene expressed a tetrameric form that has substantially higher fluorescence than the monomeric form [22, 23]. In addition to the strong red fluorescent signal and relatively high RLUC, the TK activity is also preserved in the aggregation-prone p3G TF protein. This provides evidence that the aggregation of p3G TF protein might not severely inhibit the function of the individual proteins in this TF reporter. The red fluorescence of the p3R- and p3H-expressing cells was weak and easily scattered and attenuated by the surrounding tissues* in vivo*. The* fl *gene expression can be successfully monitored* in vivo,* with 4-fold higher FLUC activity in the p3H-expressing cells than in the p3R-expressing cells indicating that the FLUC activity in TF gene constructs is preserved and is sufficient for* in vivo* bioluminescence imaging.

In the current work we clearly demonstrated the differences of tTKSR39 activity among three TF gene constructs by* in vitro* cellular uptake, but the differences in ttksr39 gene expression (1.25- to 1.4-folds) are too small to be discriminated by* in vivo* PET imaging. The individual differences of mice and the transfection efficiency contributed by different gene constructs may mask the small differences in tTKSR39 activity. Although it seems attractive to perform multiple imaging modalities with p3G TF construct, which retained the most activities of each fusion partners among all three TF constructs, the following criteria are in favor of using* fl* for BLI instead of* rl*: (1) in* rl*, the tissue penetration of light is limited due to emissions peaking at 480 nm; (2) the multidrug resistant MDR1 p-glycoprotein (PgP) which overexpresses in cancer cells [35] can alter the transport of coelenterazine (the RLUC substrate), resulting in a decreased bioluminescence signals; (3) while injected with i.p., the autoluminescence contributes to high background [36]; (4) coelenterazine is more expensive than D-luciferin (FLUC substrate). We also noted that the origin/source of* fl* reporter gene used by Ray et al. to construct the first and second generation TF gene constructs were different [13]. The nucleotide sequence of a fusion partner gene can influence the structure and stability of TF mRNA and further affect the efficiency of TF protein translation. It may explain why Ray et al. chose the* wt.tk* as the fusion partner in their second generation TF construct, even though the enzymatic affinity of tTKSR39 mutant to acycloguanisines is higher than that of the wt.TK. In order to eliminate any undesired effect resulting from certain fusion partners of uncertain origin/source, we used commercially available, codon optimized fusion partner genes to construct the p3G, p3R, and p3H. Thus, the different efficacies between p3R and p3H fusion protein demonstrated in this study resulted solely from the orientation of the genes in the reporter construct.

Our results demonstrated a decreased tTKSR39 activity in all fusion constructs. Longer spacer between the two fusion partner genes might improve the function of each protein [9]. Truncation of the first 135bp of the native* tksr39* coding sequence shortened the distance between* fl* and* ttksr39* genes. Thus, a longer spacer composed of small flexible amino acids, such as poly-glycine, or the use of the minimally truncated* tksr39* gene (only with deletion of N-amino terminal nuclear localization signal) instead of* ttksr39* can be considered as another option for the next generation of TF gene. Moreover, human codon optimization could be implemented to alter the NLS and the cryptic testicular promoter regions of the full-length tksr39 for applications in transgenic mice. Such kind of modification can remove the high frequency of CpG island in tksr39 gene and eliminate the CpG methylation associated with transcriptional silencing, resulting in high-yield and long-term gene expression of* tk* [35]. Several new* DsRed* variant genes, such as* mCherry *or* mRaspberry,* could also be used for the new generation of TF genes, which will benefit from far-red emissions spectra and little overlap with GFP and YFP [37].

In conclusion, we have developed an optimized construct of triple reporter gene for multimodality molecular imaging* in vitro* and* in vivo*. The* DsRedm-fl-ttksr39* triple reporter gene is less cytotoxic, more effective, and sensitive for* in vitro* cell imaging as well as* in vivo* animal imaging using fluorescence, bioluminescence, and PET imaging modalities, which may facilitate different fields of translational biomedical research and applications.
